# P30 Making the switch: a triple win for patients, hospitals, and the planet

**DOI:** 10.1093/jacamr/dlaf118.037

**Published:** 2025-07-14

**Authors:** Ali Taki, Marisa Lanzman

**Affiliations:** Pharmacy Department, Royal Free London NHS Foundation Trust, London, UK; Pharmacy Department, Royal Free London NHS Foundation Trust, London, UK

## Abstract

**Background:**

Antimicrobial resistance (AMR) is a major global health threat, directly causing 1.27 million deaths in 2019. In the UK, bacterial infections were linked to 87 500 deaths, with the AMR burden rising by 3.5% between 2019 and 2023. The UK’s 2024 AMR Action Plan emphasizes antimicrobial stewardship (AMS), including IV-to-oral switch (IVOS) programmes, which improve patient safety, reduce costs, and enhance efficiency. However, local barriers such as clinician awareness and uncertainty about oral alternatives persist.

**Objectives:**

This project aimed to evaluate a pharmacist-led implementation of the UK Health Security Agency (UKHSA) IVOS decision aid to optimize IV antimicrobial prescribing. The primary objective was to reduce inappropriate IV antibiotic use; the secondary objective was to decrease overall IV antibiotic consumption.

**Methods:**

This project used the Model for Improvement framework to optimize IV-to-oral antibiotic switching on acute medicine wards. A pre-intervention survey of pharmacists and physicians identified key barriers to IVOS, including low awareness of the UKHSA decision aid and uncertainty around suitable oral alternatives. Insights from the survey informed the intervention design. Daily pharmacist-led reviews were conducted for all patients on IV antibiotics, with IVOS recommendations documented using a standardized electronic proforma that incorporated the UKHSA decision aid. Two PDSA cycles were implemented: the first focused on pharmacist documentation; the second incorporated direct engagement with the medical team. Data collected at baseline and post intervention were analysed using LifeQI run charts to assess the proportion of eligible patients switched within 24 h, alongside changes in weekly IV antibiotic prescriptions.

**Results and discussion:**

Baseline data showed that only 16.67% of patients were switched from IV to oral antibiotics within 24 h of meeting eligibility. Following the first PDSA cycle of a pharmacist review and documentation, this improved to 58.33%, a 41.66% increase. Weekly IV antibiotic prescriptions fell from 117 to 100 (−14.5%). This equates to a projected Trust-wide annual saving of £843 000, the release of 13 WTE nursing staff, and a reduction of 206 776 kg CO_2_. While the second PDSA cycle introduced direct medical team engagement, it did not yield statistically significant further improvements. These results support the effectiveness of pharmacist-led IVOS reviews; however, limitations include data collection from only two acute medicine wards, small daily sample sizes that may affect percentage reliability, and variability in medical team engagement.

**Conclusions:**

This locally novel pharmacist-led IVOS initiative demonstrated significant benefits at decreasing both inappropriate IV antibiotic prescribing and total IV antibiotic usage. The intervention could help reduce AMR risk, hospital costs, and nursing workload. Wider implementation may depend on resource allocation, and future work should explore extending the intervention across other specialties to confirm reproducibility and consistency. Trialling the incorporation of pharmacy technicians under a defined protocol could also enhance cost-effectiveness and extend the intervention’s outreach.

